# Successful Treatment of Disseminated Subcutaneous Panniculitis-Like T-Cell Lymphoma with Single Agent Oral Cyclosporine as a First Line Therapy

**DOI:** 10.1155/2014/201836

**Published:** 2014-11-23

**Authors:** Nida Iqbal, Vinod Raina

**Affiliations:** Department of Medical Oncology, Dr. B. R. A. Institute Rotary Cancer Hospital, All India Institute of Medical Sciences, New Delhi 110029, India

## Abstract

Subcutaneous panniculitis-like T-cell lymphoma (SPTL) is a rare cutaneous neoplasm of mature cytotoxic T-cells. Currently there are no standardized therapies for SPTL; however good responses have been seen with chemotherapy regimens generally employed for B-cell lymphomas. Cyclosporine, an immunosuppressant, has shown good responses in relapsed/refractory SPTL; however its use in first line setting is not well established. We, herein, describe a 22-year-old girl with disseminated SPTL who attained complete clinical remission with single agent oral cyclosporine used as a first line therapy.

## 1. Introduction

Subcutaneous panniculitis-like T-cell lymphoma (SPTL) is a type of T-cell lymphoma with clinicopathologic features simulating panniculitis and associated with an aggressive clinical course [1]. There are two subtypes, TCR alpha/beta and TCR gamma/delta [2]. While TCR alpha/beta generally have a CD4−, CD8+, CD56− phenotype and a favorable prognosis, TCR gamma/delta typically have a CD4−, CD8− T-cell phenotype with frequent coexpression of CD56 and a poor prognosis [3]. SPTL most commonly affects patients in 4th decade of life with a female predominance [4]. Patients present with subcutaneous nodules or plaques most commonly on extremities and trunk that, on pathologic evaluation, demonstrate cellular infiltrates in the subcutaneous fat, generally with sparing of the overlying epidermis. Metastatic disease is very rare. Clinical and systemic symptoms are nonspecific. There is no standardized treatment for SPTL [4]. CHOP-like chemotherapy is generally used as an initial therapy. Cyclosporine, an immunosuppressant, has shown activity in relapsed/refractory SPTL [5, 6]. However, it is still not clear whether cyclosporine as a single agent is able to induce remissions in patients with disseminated SPTL and the question of its use as first line therapy remains unanswered.

In this report, we describe complete clinical remission of disseminated SPTL (TCR alpha/beta) in a 22-year-old girl with single agent oral cyclosporine used as a first line therapy.

## 2. Case Report

**Figure 1:**
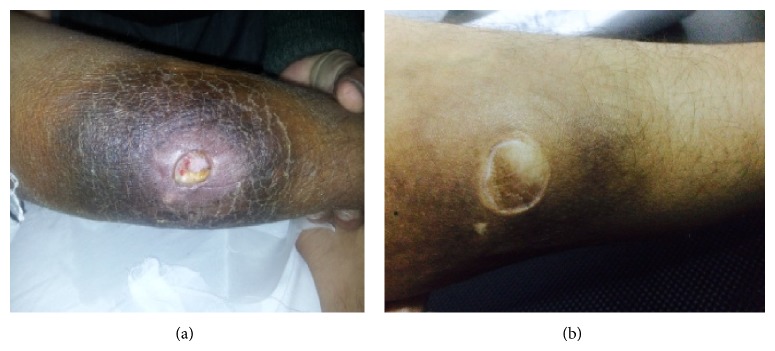
(a) Ulcerative lesion with surrounding hyperpigmented area over left calf before treatment. (b) Healed lesion 18 months after completion of therapy.

A 22-year-old girl presented with the complaints of intermittent fever, decreased appetite, and progressively increasing nodular swellings over both cheeks, lower back, both gluteal areas, and lower limbs. On physical examination, the patient was febrile with ECOG performance status of 2. There was mild hepatosplenomegaly with no peripheral lymphadenopathy. Skin examination revealed nodular swelling 3-4 cm over both cheeks and 2-3 cm swellings over lower back, both gluteal areas, and right thigh. There was an ulcerative lesion approximately 3 cm over left calf with surrounding hyperpigmented area (Figure 1(a)). The laboratory investigations revealed haemoglobin level of 8.7 g/dL, white blood cell count of 4.1 × 10^9^/L, platelet count of 275 × 10^9^/L, serum LDH of 228 U/L, and normal renal and liver function tests. The serologic tests for human immunodeficiency virus and hepatitis B and C were negative. Contrast enhanced CT chest and abdomen did not reveal any abnormality. PET scan revealed diffuse uptake in subcutaneous fat, left breast, liver, and spleen. Bone marrow examination was normal. There was no evidence of hemophagocytic syndrome. An excisional biopsy from the skin nodule showed septal and interstitial panniculitis with fat necrosis and atypical lymphoid cell infiltrate focally lining the adipocytes, mainly limited to subcutaneous tissue and lower dermis. Foamy histiocytes with beanbag cells were also identified in the interstitium. Immunohistochemistry analysis showed that tumor cells expressed CD3 and CD8 but were negative for CD20, CD4, and CD 56. Ki-67 was not measured. These findings were consistent with the diagnosis of subcutaneous panniculitis-like T-cell lymphoma (alpha/beta subtype).

She was started on oral cyclosporine 4 mg/kg/d as a single agent. Patient responded well with resolution of all systemic manifestations of disease within two weeks of initiation of therapy. All skin nodules resolved completely within two months. She was continued on same dose of cyclosporine for next 5 months and finally the drug was tapered over 1 month. She tolerated the therapy well except for mild hirsutism. PET scan done at 6 months of treatment showed complete resolution of all subcutaneous nodules with mild uptake in left calf. Currently, the patient is in clinical remission 18 months after completion of therapy (Figure 1(b)).

## 3. Discussion

Subcutaneous panniculitis-like T-cell lymphoma (SPTL) was first described by Gonzalez et al. in 1991 [1] and defined as a distinct entity by the World Health Organization (WHO) classification in 2001 [7, 8]. SPTL is a cutaneous condition and metastatic disease or visceral involvement is uncommon. CD56 is an important marker in T-cell lymphomas as CD56 positive tumors have worse outcome in view of disseminated disease and hemophagocytosis [9]. Only cases with TCR gamma/delta phenotype (mostly CD56 positive) tend to have metastasis to various organs, including lungs, liver, kidneys, and the central nervous system. Hemophagocytic syndrome (HPS) characterized by fever, pancytopenia, hepatosplenomegaly, and coagulopathy is most commonly seen in TCR gamma/delta phenotype and associated with aggressive outcome [10]. TCR alpha/beta phenotype is generally CD56 negative and associated with favourable outcome. The overall five-year survival rate for TCR alpha/beta exceeds 80%; however, in the presence of HPS, it reduces to less than 50%. In cases of TCR gamma/delta, the five-year survival rate is less than 20% in either group [4].

Due to rarity of disease, no standardized therapy for SPTL currently exists. For indolent local disease, local radiotherapy can be used as an effective treatment modality. For indolent disease with a more generalized distribution, systemic biologic agents may be used, such as bexarotene and interferon, as well as low-dose chemotherapy with agents such as methotrexate. For aggressive presentations, doxorubicin-based therapies are most commonly used, with overall complete or partial remission rates of 50%. Fludarabine-based chemotherapies have shown an overall remission rates of more than 70% in a few case reports [11, 12]. A case of SPTL with HPS resistant to CHOP regimen achieved complete remission after combination chemotherapy using BFM-90 protocol [11]. High-dose chemotherapy followed by stem cell transplantation have been reported to produce the highest response rates [13, 14].

Immunosuppressive therapy with steroids and cyclosporine have shown good results in the treatment of relapsed/refractory SPTL in a few case reports [5, 6]. Cyclosporine, a calcineurin inhibitor, is a potent immunosuppressant. The mechanism of action of cyclosporine in SPTL is downregulation of cytokines. Despite its magical effects in a few cases of relapsed/refractory SPTL, we could not find any report of its use in upfront setting. Our case is the first of its kind in which upfront use of cyclosporine was able to maintain durable remission in patient with disseminated SPTL.

## 4. Conclusion

Because of lack of standard treatment to SPTL, cyclosporine may be a good option as a first line therapy even in patients with disseminated disease due to lack of side effects and ease of administration. However, its benefit in patients with TCR gamma/delta phenotype needs to be confirmed by further studies.
